# Construction and Validation of a Novel Prognostic Signature for Intestinal Type of Gastric Cancer

**DOI:** 10.1155/2021/5567392

**Published:** 2021-08-12

**Authors:** Fan Zhang, Ewetse Paul Maswikiti, Yucai Wei, Wenzhang Wu, Yumin Li

**Affiliations:** ^1^Department of General Surgery, Lanzhou University Second Hospital, 82 Cuiyingmen, Lanzhou, Gansu Province 730030, China; ^2^Key Laboratory of Digestive System Tumors of Gansu Province, Lanzhou, Gansu Province 730030, China; ^3^Department of Oncology Surgery, Lanzhou University Second Hospital, 82 Cuiyingmen, Lanzhou, Gansu Province 730030, China

## Abstract

**Background:**

Intestinal type of gastric cancer (IGC) is the largest subtype of gastric cancer (GC) by Lauren classification. The purpose of this present study was to construct a prognostic signature for IGC patients, based on the high-grade dysplasia (HGD) and IGC tissues, to improve and enhance the prognostic accuracy.

**Methods:**

The microarray datasets and associated clinical characteristics of HGD and IGC were obtained from the Gene Expression Omnibus (GEO) database. Based on the differential expression analysis between HGD and IGC, the prognostic-related differential expression genes (DEGs) were identified in a training set by univariate COX regression analysis. The least absolute shrinkage and selection operator (LASSO) regression was used to construct an optimal prognostic signature. The enrichment analysis was performed by using Gene Set Enrichment Analysis (GSEA). The performance of the nomogram was assessed by the calibration curve and concordance index (C-index). The results were validated by using a testing set.

**Results:**

We identified 35 prognostic-related DGEs in the training set. The nine-gene signature was established by LASSO analysis. The nine-gene signature was an independent risk factor in both the training and testing sets. The areas under the curve (AUC) values of receiver operating characteristic (ROC) analysis were 0.733 and 0.700 for the training and testing sets, respectively. In GSEA analysis, the gene expression in high-risk group was enriched in hedgehog signaling, epithelial mesenchymal transition, and angiogenesis. The nomogram for IGC showed good performance with C-index of 0.81 (95% CI: 0.76-0.86) and 0.70 (95% CI: 0.63-0.77) in the training and testing sets, respectively.

**Conclusion:**

We identified and verified a nine-gene signature for the prognostic prediction of IGC patients, which might identify subgroups of IGC patients and select more suitable therapeutic options.

## 1. Introduction

Gastric cancer (GC) is the fifth most common cancer and the third leading cause of cancer-related deaths worldwide, with 27,510 incidences and 11,410 mortalities since 2019 [1]. It is desirable to explore accurate prognostic models which could identify the subset of patients with a high risk for death and prompt to give those timely treatments. A number of previous studies have established the different types of prognostic signatures for GC. Several studies have demonstrated that associated gene signatures for GC patients to predict the prognosis have been identified, including six-gene signature, five-gene signature, 24-long noncoding RNA (lncRNA) signature, and 14-lncRNA signature [2–5].

Based on Lauren classification, GC can be divided into intestinal-type, diffuse-type, and mixed-type [6]. The tumorigenesis of IGC primarily results from environmental factors, such as *Helicobacter pylori* (H. pylori) infection, and is mostly associated with geriatric patients [7, 8]. Diffuse-type of GC (DGC) was more commonly observed in younger individuals with worse prognosis [9]. The carcinogenesis of IGC is a complicated multistep process, including chronic atrophic gastritis (CAG), intestinal metaplasia (IM), low-grade dysplasia (LGD), high-grade dysplasia (HGD), and eventually carcinoma [10]. The carcinogenic pathways of DGC are mostly attributed to genomic aberrations and are less associated with environmental factors and chronic inflammatory cascade [11, 12]. Furthermore, Jinawath et al. indicated that IGC and DGC had different mechanisms underlying gastric carcinogenesis by screening the gene expression profile [13]. Therefore, there is a great need to construct a novel prognostic signature for IGC patients.

HGD is an obvious premalignant lesion, requiring aggressive treatments such as endoscopic interventions [14]. A meta-analysis illustrated that the progression rate of the patients from HGD to GC was 16 times higher than those from LGD to GC [15]. In our present study, we conducted gene differential expression analysis of multiple gene expression profiles between HGD and IGC to determine the potential mechanisms in this progression. Subsequently, we identified prognostic-related differential expression genes (DEGs) between HGD and IGC. The prognostic model was constructed based on prognostic-related DEGs and was validated for IGC patients in both the training and testing sets. Finally, we integrated the prognostic signature and clinical factors to establish a clinical nomogram and assessed the accuracy in predicting the survival rates of IGC patients.

## 2. Materials and Methods

### 2.1. Microarray Datasets

All microarray data were downloaded from the Gene Expression Omnibus (GEO) database (http://www.ncbi.nlm.nih.gov/geo/). We identified and downloaded the microarray data (GSE55696, GSE87666, GSE130823), which enrolled HGD and IGC samples [4, 16, 17]. Moreover, the six independent microarray data of IGC who underwent gastrectomy were included in the current study, including GSE26901, GSE26899, GSE66229, GSE26253, GSE29272, and GSE13861 [18, 19]. The detailed information of each dataset was listed in Table 1. The clinical information of IGC patients was collected from corresponding literature. We randomly and equally divided the IGC patients into training and testing sets for the validation.

### 2.2. Data Processing

**Figure 1:**
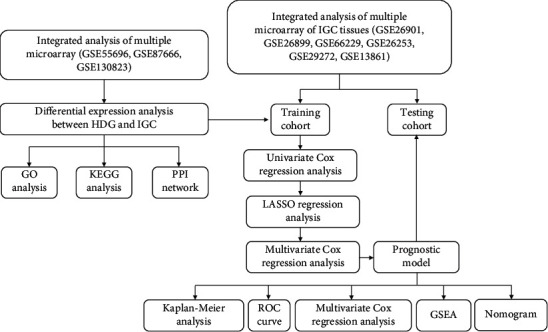
An overview of the experimental design and main procedures.

The workflow of the current study is shown in Figure 1. The raw CEL format files or gene expression matrices were normalized by using normalize Between Arrays function of limma package in R (https://bioconductor.org/biocLite.R). To reduce noise and batch effects in the microarray gene expression data, batch normalization was performed by using sva and limma package in R. Differential expression genes (DEGs) between HGD and IGC were selected by using |log2 fold change (FC)|>0.58 and false discovery rate (FDR) <0.05 in R.

### 2.3. Enrichment Analysis of DEGs between HGD and IGC

Gene Ontology (GO) terms and Kyoto Encyclopedia of Genes and Genomes (KEGG) analysis were performed using stringi and ggplot2 packages in R. GO term contains three domains: biological process (BP), cellular component (CC), and molecular function (MF). FDR <0.05 was considered statically significant. The top 10 terms of each domain for GO analysis and the top 30 terms for KEGG analysis were presented.

### 2.4. Protein-Protein Interaction (PPI) Network Analysis of DEGs

The detailed description of PPI network construction has been introduced in our previously published article [20]. The PPI network construction was analyzed using the STRING database (http://string-db.org/) [21]. The PPI pairs with an interaction score >0.7 were considered significant. The PPI network was constructed by using Cytoscape 3.6.1. Moreover, subclusters in the PPI network were identified by using the Molecular Complex Detection (MCODE) plug-in of Cytoscape [22]. The selection criteria for the subclusters were as follows: MCODE score ≥ 6, degree cutoff = 2, node score cutoff = 0.2, and k − score = 2. The hub genes in the PPI network were selected by calculating the degree with the cytoHubba plug-in of Cytoscape [23].

### 2.5. Identification of Prognostic-Related Genes

To evaluate the prognostic values of the DEGs between HGD and IGC, a univariate Cox regression analysis was performed in the training set by using the survival package in R. Subsequently, the least absolute shrinkage and selection operator (LASSO) regression was used to build an optimal prognostic signature for IGC patients by glmnet package in R. The prognostic risk score for overall survival (OS) was calculated based on the gene expression weighted by the regression coefficient in the multivariate Cox regression analysis. Receiver operating characteristic (ROC) curves were used to evaluate the accuracy of the prognostic value in IGC by using survivalROC package in R.

### 2.6. Gene Set Enrichment Analysis

The enrichment analysis between high-risk and low-risk cohorts of IGC patients was performed by using a Gene Set Enrichment Analysis (GSEA, http://www.broadinstitute.org/gsea/index.jsp) v3.0 software [24, 25]. GSEA was selected annotated gene sets h.all.v7.2.symbols.gmt. Enrichment scores (ES) and normalized enrichment scores (NES) were calculated using permutation testing (1000 permutations). NES with *P* values < 0.05 and FDR <25% were considered significantly enriched.

### 2.7. Construction and Assessment Nomogram for IGC Patients

The variates of IGC were used to construct a predicting nomogram for IGC patients by using the training set. The nomogram was constructed by using survival and rms packages in R. Harrell's concordance index (C-index) was used to estimate the prognostic efficacy of the predictive models.

## 3. Results

### 3.1. Identification of DEGs between HGD and IGC

We integrated multiple microarray datasets, containing 43 HGD and 41 IGC tissues. After differential expression analysis, 637 DEGs were identified between HGD and IGC, including 602 upregulated genes and 35 downregulated genes.

### 3.2. Functional and Pathway Enrichment Analysis

**Figure 2:**
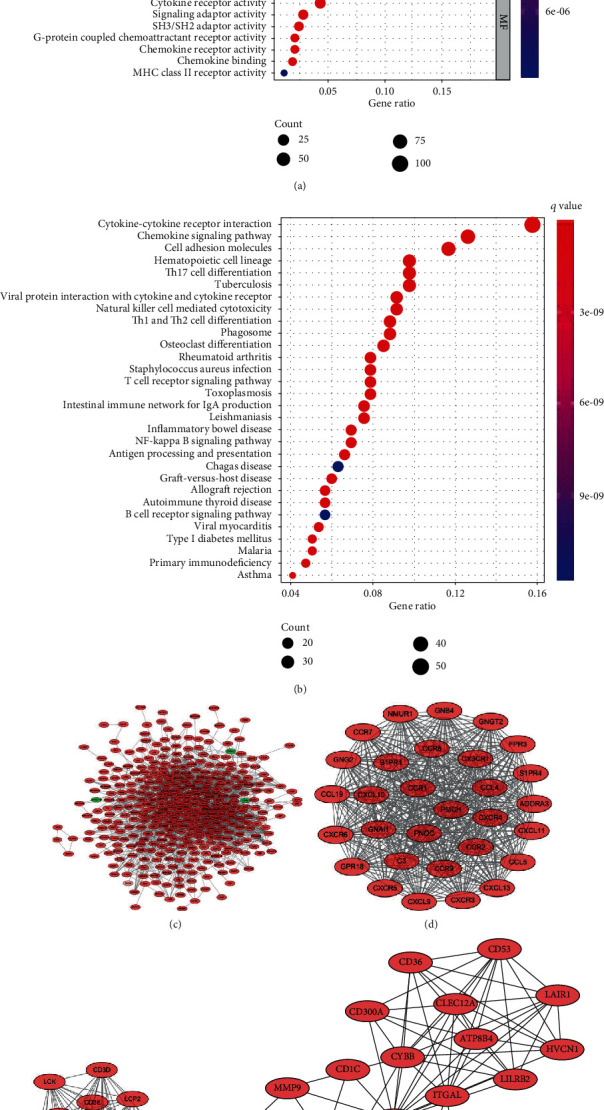
The Gene Ontology (GO) terms, Kyoto Encyclopedia of Genes and Genomes (KEGG) analysis, protein-protein interaction (PPI) network of DEGs between HGD and IGC. (a) Top 10 GO terms in biological process (BP), cellular component (CC), and molecular function (MF) domains. (b) Top 30 pathways of KEGG enrichment analysis. (c) PPI network of DEGs between HGD and IGC was constructed by using Cytoscape. (d–f) The modules were identified by using the MCODE algorithm.

To better understand the potential functions of DEGs between HGD and IGC, GO and KEGG analyses were performed. The results in the BP category were mainly enriched in immune-associated terms, such as T cell activation, regulation of lymphocyte activation, and leukocyte cell-cell adhesion (Figure 2(a)). The significantly enriched CC term included the external side of the plasma membrane, receptor complex, and endocytic vesicle (Figure 2(a)). Furthermore, cytokine receptor binding, cytokine activity, and cytokine binding were primarily enriched in the MF category (Figure 2(a)). The KEGG enrichment analysis results revealed that the primary pathways were enriched in cytokine-cytokine receptor interaction, chemokine signaling pathway, and cell adhesion molecules (Figure 2(b)).

### 3.3. Construction of PPI Network and Subclusters

PPI network was visualized by using Cytoscape (Figure 2(c)). According to the MCODE plug-in, three modules were identified in the PPI network (Figures 2(d)–2(f)). The KEGG enrichment analysis showed that genes of the module in Figure 2(d) were mainly enriched in the chemokine signaling pathway, cytokine-cytokine receptor interaction, and Toll-like receptor signaling pathways. Cell adhesion molecules, human T-cell leukemia virus type 1 (HTLV-I) infection, and T cell receptor signaling pathway were significantly enriched in the module, presented in Figure 2(e). In the module (Figure 2(f)), the analysis also revealed significant enrichment of malaria, transcriptional misregulation in cancer, and hematopoietic cell lineage pathways. After calculating the degree of each gene in the PPI network by cytoHubba, the top 10 hub genes of the PPI network were PTPRC, IL6, LCK, ITGAM, TNF, CCR7, GNG2, CCR5, CXCR4, and CD3G.

### 3.4. Characteristics of IGC Patients

A total of 503 IGC patients were enrolled in the current study, including 126 (25.05%) females and 377 (74.95%) males. The detailed information of IGC patients is presented in Table 2. The IGC patients with stage I, II, III, and IV accounted for 17.30%, 29.03%, 37.17%, and 16.50%, respectively. The survival data of 7 IGC patients could not be acquired. After removing 7 patients without prognostic information, the training set and the testing set contained 248 IGC patients, equally. The clinical characteristics were not significantly different between the training and testing sets (Table 3).

### 3.5. Assessment of the Prognostic Values of DEGs and Construction of a Prognostic Signature for IGC Patients

**Figure 3:**
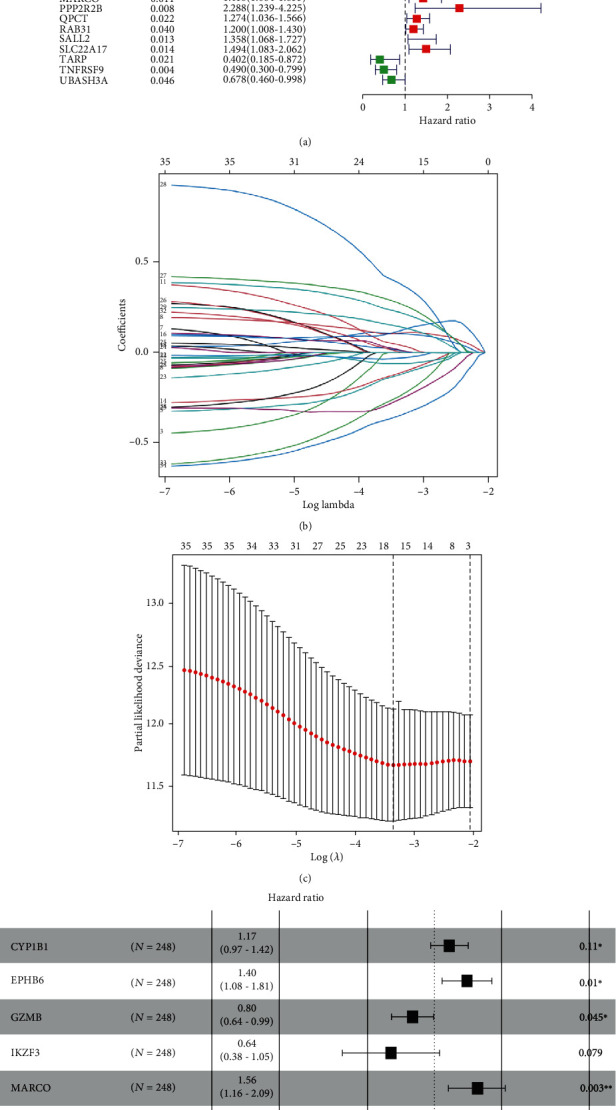
Screening the prognostic-related DEGs and construction of a prognostic signature for IGC patients. (a) Forest map revealed that 35 prognostic-related genes were identified by using univariate Cox regression analysis. (b, c) Based on LASSO analysis, a total of 9 genes were screened. (d) Forest plot of multivariate Cox regression analysis for 9 prognostic-related genes.

**Figure 4:**
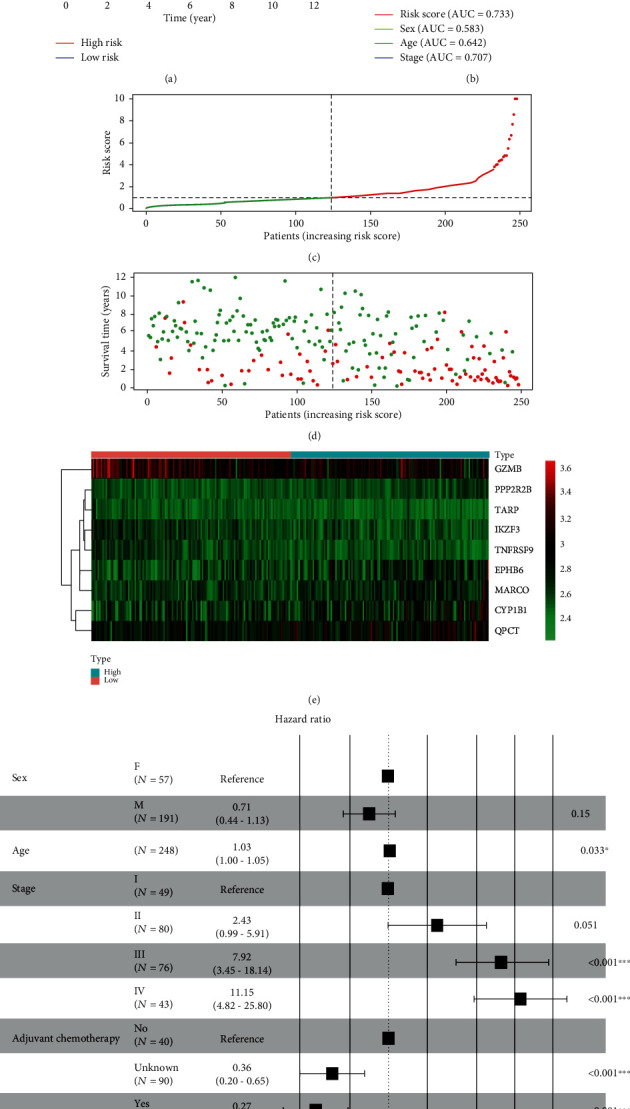
Risk score analysis of nine-gene signature for IGC patients in the training cohort. (a) Kaplan-Meier analysis showed the IGC patients in high-risk groups had a shorter OS than those in low-risk groups (*P* = 4.85 × 10^−7^). (b) ROC analysis showed that the AUC of risk score, sex, age, and stage were 0.733, 0.583, 0.642, and 0.707, respectively. (c) The distribution of risk score for each individual. (d) Survival status for each IGC patients. (e) Heatmap for the nine prognostic-related genes between high-risk and low-risk groups. (f) Multivariate Cox analysis found that the nine-gene signature was an independent prognostic indicator for IGC patients.

To screen the genes which were related to prognosis, a total of 35 DGEs were identified as prognostic-related genes by using univariate Cox regression analysis. The forest map presented that the hazard ratio and *P* value of each prognostic-related gene (Figure 3(a)). Subsequently, a total of 9 genes were screened by LASSO analysis, including cytochrome P450 family 1 subfamily B member 1 (CYP1B1), EPH receptor B6 (EPHB6), granzyme B (GZMB), IKAROS family zinc finger 3 (IKZF3), macrophage receptor with collagenous structure (MARCO), protein phosphatase 2 regulatory subunit Bbeta (PPP2R2B), glutaminyl-peptide cyclotransferase (QPCT), TCR gamma alternate reading frame protein (TARP), and TNF receptor superfamily member 9 (TNFRSF9), presented in Figures 3(b) and 3(c). The risk model was constructed by a multivariate Cox regression analysis, presented in Figure 3(d). Risk score = (0.158 × expression level of CYP1B1) + (0.337 × expression level of EPHB6) + (−0.225 × expression level of GZMB) + (−0.452 × expression level of IKZF3) + (0.445 × expression level of MARCO) + (0.765 × expression level of PPP2R2B) + (0.254 × expression level of QPCT) + (−0.728 × expression level of TARP) + (−0.576 × expression level of TNFRSF9). After dividing the patients into high-risk and low-risk groups based on median risk score, the Kaplan-Meier survival analysis for the training set showed that the IGC patients with high-risk scores had significantly reduced the OS rate compared to those with low-risk scores (*P* = 4.85 × 10^−7^, Figure 4(a)). To validate the accuracy of the risk model, ROC analysis for risk score, sex, age and stage indicated that the areas under the ROC curves (AUC) were 0.733, 0.583, 0.642, and 0.707 for the training set, respectively (Figure 4(b)). In addition, the distribution of risk scores, survival status, and expression values of 9 DEGs was presented in Figures 4(c)–4(e). The results illustrated that the IGC patients with high-risk scores had a lower survival rate than those with low-risk scores. Of note, multivariate COX regression analyses demonstrated that the low risk score (HR = 0.41, 95% CI: 0.26-0.63, *P* < 0.001), age (HR = 1.03, 95% CI: 1.00-1.05, *P* = 0.033), stage III (HR = 7.92, 95% CI: 3.45-18.14, *P* < 0.001), stage IV (HR = 11.15, 95% CI: 4.82-25.80, *P* < 0.001), and adjuvant chemotherapy (HR = 0.27, 95% CI: 0.15-0.48, *P* < 0.001) were independent prognostic factors, as shown in Figure 4(f).

### 3.6. Validation of Prognostic Model for IGC Patients

**Figure 5:**
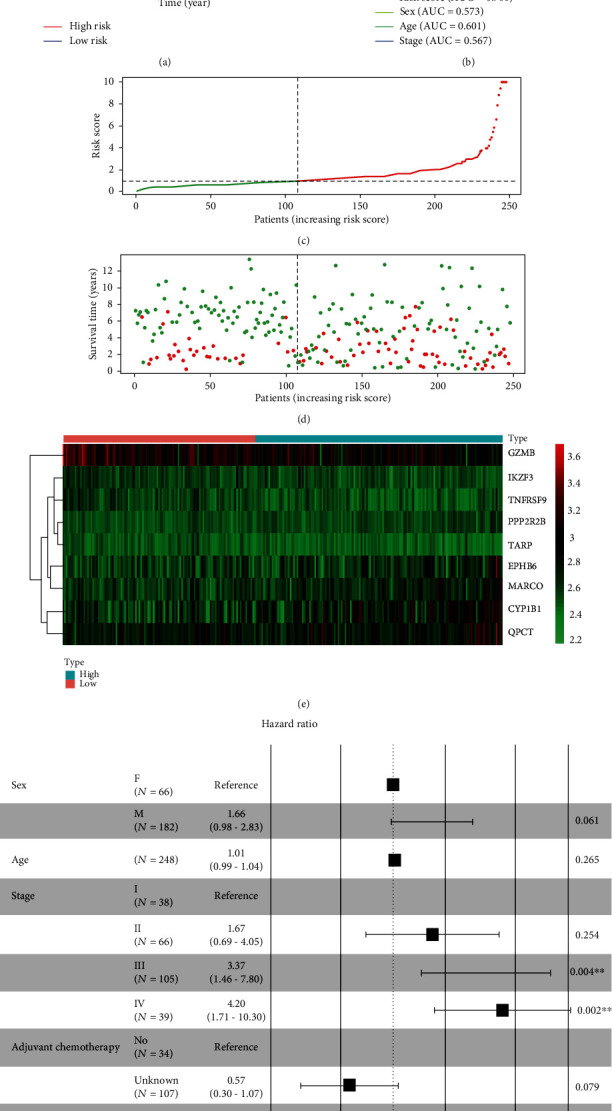
Validation of the nine-gene signature for IGC patients in the testing cohort. (a) Kaplan-Meier analysis showed that IGC patients with high-risk scores had a shorter OS than those with low-risk scores (*P* = 1.445 × 10^−3^). (b) ROC analysis showed that the AUC of risk score, sex, age, and stage in the testing group were 0.7, 0.573, 0.601, and 0.567, respectively. (c–e) An overview of the survival status, the distributions of the risk score for each patient, and heatmaps for nine-gene signature in the testing group. (f) Multivariate Cox analysis for IGC patients in the testing cohort also identified that the nine-gene signature was an independent risk factor.

To validate the prognostic value of the risk scoring model, the survival rate of IGC patients in the testing set was consistent with those in the training set by using Kaplan-Meier survival analysis (*P* = 1.45 × 10^−3^, Figure 5(a)). ROC curve analysis showed that the AUC of risk score, sex, age, and stage in the testing set were 0.700, 0.573, 0.601, and 0.567, respectively (Figure 5(b)). In the testing set, we found out that the patients in the low-risk group had a significantly better OS than those in the high-risk group, which was in consistent with the training set (Figures 5(c)–5(e)).

### 3.7. Exploration of Enriched Pathways between High-Risk and Low-Risk Cohorts

**Figure 6:**
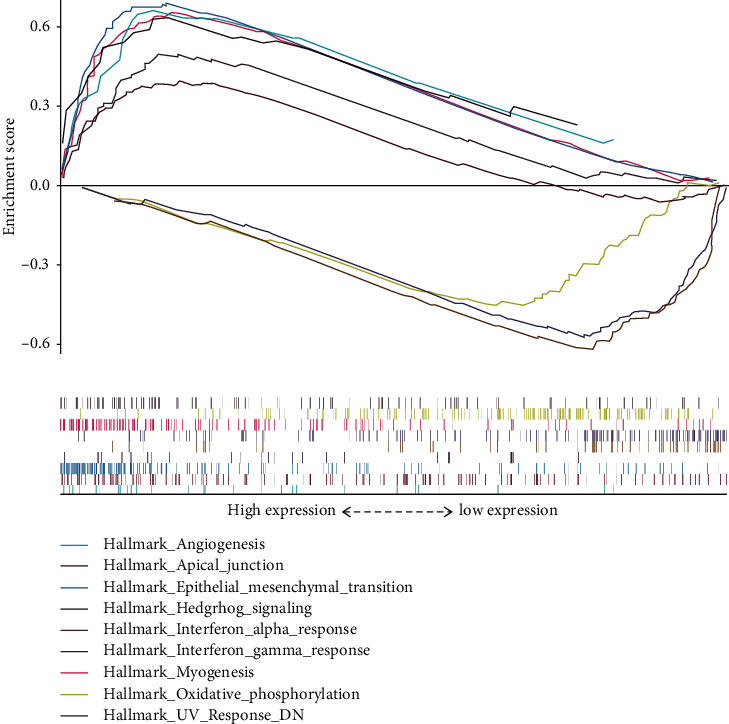
The Hallmark enrichment analysis between high-risk and low-risk groups by Gene Set Enrichment Analysis (GSEA).

In order to further elucidate the potential mechanisms, GSEA with hallmark gene sets was conducted between high-risk and low-risk cohorts (Table 4). The gene expression in the high-risk group was enriched in myogenesis, hedgehog signaling, epithelial-mesenchymal transition (EMT), ultraviolet (UV) response down (DN), angiogenesis, and apical junction (Figure 6). Furthermore, the low-risk group was enriched in oxidative phosphorylation, interferon gamma response, and interferon alpha response (Figure 6).

### 3.8. Construction of Nomogram for IGC Patients

**Figure 7:**
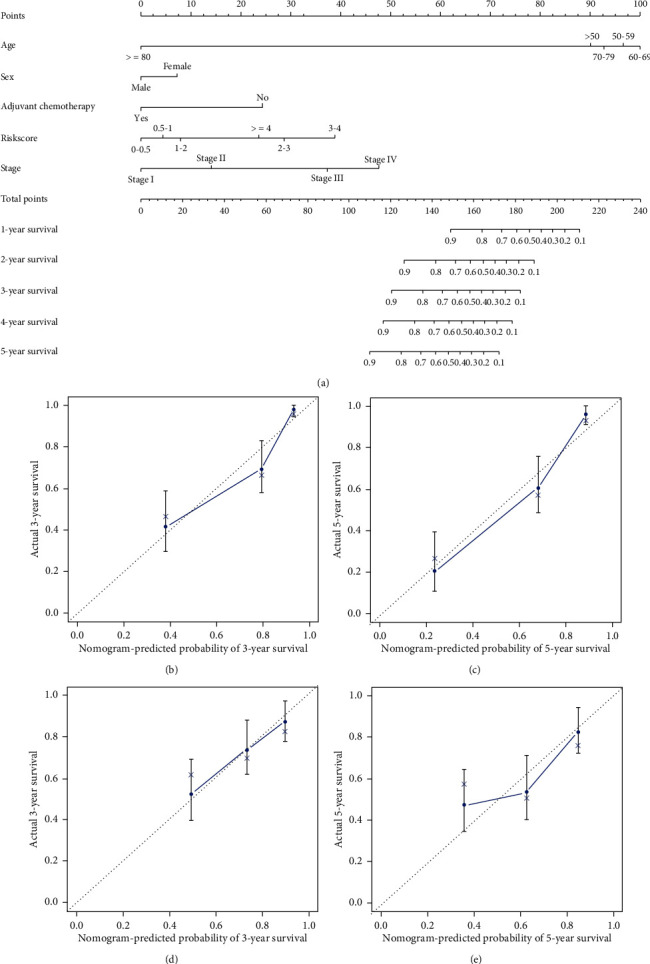
Nomogram and the calibration curves of the prognostic model for IGC patients. (a) Construction of nomogram for IGC patients who underwent gastrectomy. The calibration curves for predicting the overall survival for IGC patients at 3 years (b) and 5 years, (c) in the training group and at 3 years, (d) and 5 years (e) in the testing group.

A nomogram for OS was constructed by age, sex, adjuvant chemotherapy, risk score, and stage (Figure 7(a)). The C-index calculated in the training set for OS prediction was 0.81 (95% CI: 0.76-0.86), indicating the suitability of a new predictive model for IGC patients. In the testing set, the C-index of the nomogram for predicting OS was 0.70 (95% CI: 0.63-0.77). The predictions on the three- and five-year survival probability for IGC patients in the training and testing sets are shown in calibration plots, respectively (Figures 7(b)–7(e)).

## 4. Discussion

It has been reported that IGC constitutes the largest proportion of GC by Lauren classification [26]. The tumorigenesis of IGC is a complex and complicated process, and HGD is a key precancerous lesion with a specific pathologic characteristic. Therefore, the selection of HDG and IGC to do further analysis was more reasonable. In the present study, we identified 637 DEGs after a comparison of 43 HGD tissues and 41 IGC tissues. The pathways and GO term enrichment analyses may suggest the potential mechanisms during the tumorigenesis from HGD to IGC. We found out that immune-related and inflammation-related pathways, like the T cell activation pathway, were significantly enriched. More evidence showed that chronic inflammation may induce progression from CAG to IM that may increase the likelihood of GC [27]. Moreover, the maintenance of IM with chronic inflammation and progression of spasmolytic polypeptide expressing mucosa (SPEM) to IM by promoting proinflammatory signals can predispose an individual to induce dysplasia [28]. Blocking T cell activation during the H. pylori infectious process may inhibit and reverse established preneoplastic lesions [29]. Coincidentally with our study, T cell activation may play an important role in gastric tumor carcinogenesis.

The KEGG analysis showed that some potential significant signal pathways were screened in the progression from HGD to IGC. We found out that Th1 and Th2 cell differentiation pathway was significantly enriched in the progression from HGD to IGC. Similarly, Ren et al. found that the significant differences between gastritis with and without cancer and dysplasia indicated a shift from a Th1 to a Th2 helper cell pattern of cytokine secretion [30]. In addition, we also detected the enrichment of the Th17 cell differentiation pathway. The number of Th17 cells in peptic ulcer patients with H. pylori infection was significantly higher than those in the patients with gastritis [31]. The results indicated that the predominant Th17 cell responses may have been involved in the gastric tumorigenesis with H. pylori infection.

Through Cox and LASSO regression analysis, we constructed a predictive risk model for IGC patients based on nine prognostic-related DEGs. After dividing the IGC patients into high-risk and low-risk groups, Kaplan-Meier analysis and ROC curves indicated that the model of training and validation cohorts both had a good performance. To the best of our knowledge, this is the first time to construct a nomogram for predicting the OS of IGC patients who underwent gastrectomy. Moreover, the calibration curves and C-indexes showed good concordance. Zhang et al. reported that the five-gene signature for GC patients achieved a higher C-index in OS than the six-gene and 24-lncRNA signatures [4]. Most importantly, our nine-gene signature for IGC patients showed a higher C-index than the five-gene signature for GC.

To further explore the potential molecular mechanisms between high-risk and low-risk groups based on the risk score, GSEA in the Hallmark pathway database was performed, whereby the results illustrated that the high-risk cohort was significantly enriched in hedgehog signaling, EMT, angiogenesis, and apical junction pathways. The hedgehog signaling pathway plays a critical role in gastric development, homeostasis, and tumorigenesis [27, 32]. Activation of the EMT pathway could induce gastric epithelial cells to turn into mesenchymal cells, causing tumor metastasis by attenuating a cell-cell adhesion and alteration of cell polarity [33]. Angiogenesis is a hallmark of solid tumor development and also an important prerequisite for tumor growth and metastasis [34]. Overall, the IGC patients with high-risk score were characterized by a high invasion and fast growth. Therefore, antiangiogenic therapies, such as Bevacizumab and Ramucirumab, may be more suitable for high-risk IGC patients based on our risk score.

The strength of our study is that, to our knowledge, it represents a novel gene signature evaluating the prognostic value for IGC patients. However, there are still several limitations in the present study. First, we only incorporated the microarray datasets which included the clinical characteristics and survival data for IGC patients. Second, the data regarding H. pylori infection status is unknown.

## 5. Conclusion

In the present study, we identified and verified a novel nine-gene signature for the prognostic prediction of IGC patients, which might identify subgroups of IGC patients with different risk scores. The nomogram could accurately predict the prognosis for IGC patients. The nine-gene signature may help to select more suitable therapeutic options for different subgroups of IGC patients.

## Figures and Tables

**Table 1:** Details of microarray datasets in Gene Expression Omnibus database.

Dataset	Platform	Sample size	Gender
GSE55696	GPL6480	39 (HGD: 20, IGC: 19)	F: 11, M: 28
GSE87666	GPL17077	15 (HGD: 9, IGC: 6)	F: 4, M: 11
GSE130823	GPL17077	30 (HGD: 14, IGC: 16)	F: 11, M: 19
GSE26901	GPL6947	82 IGC	F: 28, M: 54
GSE26899	GPL6947	59 IGC	F: 11, M: 48
GSE66229	GPL570	146 IGC	F: 36, M: 110
GSE26253	GPL8432	139 IGC	F: 29, M: 110
GSE29272	GPL96	58 IGC	F: 16, M: 42
GSE13861	GPL6884	19 IGC	F: 6, M: 13

HGD: high-grade dysplasia; IGC: intestinal type of gastric cancer; F: female; M: male.

**Table 2:** Clinical characteristics of intestinal type of gastric cancer from multiple GEO datasets.

Characteristics	Number of IGC	Percentages (%)
Gender		
Female	126	25.05%
Male	377	74.95%
Age		
Mean (SD)	59.80 (10.31)	
≥65	174	34.59%
<65	329	65.41%
Stage		
I	87	17.30%
II	146	29.02%
III	187	37.18%
IV	83	16.50%
Adjuvant therapy		
Yes	225	44.73%
No	74	14.71%
Unknown	204	40.56%
Status		
Death	186	36.98%
Alive	310	61.63%
Unknown	7	1.39%

**Table 3:** Comparison of clinical characteristics between the training and testing sets.

Characteristics	Training set	Testing set	*P* value
	Number (%) or mean ± SD	Number (%) or mean ± SD	
Sex			0.349
Male	191 (77.0%)	182 (73.4%)	
Female	57 (23.0%)	66 (26.6%)	
Age (year)	60.16 ± 10.39	59.45 ± 10.25	0.442
Stage			0.056
I	49 (19.8%)	38 (15.3%)	
II	80 (32.3%)	66 (26.6%)	
III	76 (30.6%)	105 (42.3%)	
IV	43 (17.3%)	39 (15.7%)	
Adjuvant chemotherapy			0.288
Yes	118 (47.6%)	107 (43.1%)	
No	40 (16.1%)	34 (13.7%)	
Unknown	90 (36.3%)	107 (43.1%)	
Death			0.578
Yes	152 (61.3%)	158 (63.7%)	
No	96 (38.7%)	90 (36.3%)	
Recurrence			0.063
Yes	95 (38.3%)	81 (32.7%)	
No	135 (54.4%)	134 (54.0%)	
Unknown	18 (7.3%)	33 (13.3%)	
GEO database			0.241
GSE26901	46 (18.5%)	36 (14.5%)	
GSE26899	28 (11.3%)	31 (12.5%)	
GSE66229	72 (29.0%)	74 (29.8%)	
GSE26253	75 (30.2%)	64 (25.8%)	
GSE29272	18 (7.3%)	33 (13.3%)	
GSE13861	9 (3.6%)	10 (4%)	

**Table 4:** Results of Gene Set Enrichment Analysis (GSEA) between high-risk and low-risk cohorts.

Name	ES	NES	*P* value	FDR
MYOGENESIS	0.652	1.931	0	0.019
HEDGEHOG SIGNALING	0.631	1.798	0.002	0.041
EPITHELIAL MESENCHYMAL TRANSITION	0.687	1.746	0.004	0.046
UV RESPONSE DN	0.499	1.725	0.004	0.041
ANGIOGENESIS	0.660	1.633	0.031	0.073
APICAL JUNCTION	0.393	1.489	0.028	0.160
OXIDATIVE PHOSPHORYLATION	-0.461	-1.855	0.022	0.083
INTERFERON GAMMA RESPONSE	-0.575	-1.673	0.037	0.225
INTERFERON ALPHA RESPONSE	-0.628	-1.643	0.046	0.189

ES: enrichment scores; NES: normalized enrichment scores; FDR: false discovery rate.

## Data Availability

The datasets, including GSE55696, GSE87666, GSE130823, GSE26901, GSE26899, GSE66229, GSE26253, GSE29272, and GSE13861, generated during the current study are available in the Gene Expression Omnibus (GEO) database (http://www.ncbi.nlm.nih.gov/geo/).
